# Crack growth and energy dissipation in paper

**DOI:** 10.1038/s41598-018-35500-6

**Published:** 2018-11-26

**Authors:** Maryam Hanifpour, Tero Mäkinen, Juha Koivisto, Markus Ovaska, Mikko J. Alava

**Affiliations:** Aalto University, Department of Applied Physics, PO. Box 11100, 00076 Aalto, Finland

## Abstract

Here, we follow the stable propagation of a roughening crack using simultaneously Digital Image Correlation and Infra-Red imaging. In a quasi-two-dimensional paper sample, the crack tip and ahead of that the fracture process zone follow the slowly, diffusively moving “hot spot” ahead of the tip. This also holds when the crack starts to roughen during propagation. The well-established intermittency of the crack advancement and the roughening of the crack in paper are thus subject to the dissipation and decohesion in the hot spot zone. They are therefore not only a result of the depinning of the crack in a heterogeneous material.

## Introduction

Fracture as a physics problem is a challenge since many of the features seen in an experiment on failure exhibit complex phenomena^1^. The sample response to loading is not smooth but intermittent, and cracks move likewise by avalanches and leave rough trails or crack surfaces. Understanding why and how is important both for fracture mechanics and for the fundamental reasons that give rise to such behavior. The complexity seen means that ordinary Linear Elastic Fracture Mechanics (LEFM) or the minimal theoretical approach needs to be considered with care, and the understanding of how strong materials are, and why, is quite challenging.

One recently found and developed paradigm to explain avalanches and such is the depinning of cracks, as an application of a general non-equilibrium phase transition in statistical mechanics^1–5^. The addition of disorder in the material combines with the driving external stress and the self-elasticity of the crack front to create a critical point: precisely at the disorder-dependent external stress value the crack velocity is zero excluding creep propagation. From this follows a number of important consequences like the critical strength and its dependence on sample size, the (in- and out-of-plane) self-affine roughness exponents measuring the rough cracks, the fluctuating character of the crack propagation velocity due to avalanches, and the sub-critical response or creep. Such theories have been applied with success to planar crack fronts^2^, to account for crack surface roughening^1^, and to explain the stress-dependence of the creep rate^6,7^, to mention a few.

However, fracture is a multi-scale phenomenon. Critical crack propagation describes materials with a few key extra parameters like the strength of the elastic coupling of fluctuations along the crack front, the average fracture toughness and the standard deviation as a measure of disorder^1,3^. In reality, cracks exist in an environment where several coupled phenomena may take place. Plastic deformation ahead of the crack and material weakening or “damage mechanics” due to the overloading of the material in the proximity are both often simply summarized by the presence of a Fracture Process Zone (FPZ)^8,9^. It is an open question to which degree the generalization of LEFM by depinning is applicable, and what other scenarios may be found.

Here, we study the failure of a notched material sample in the context of an elasto-plastic material (copy paper) with a FPZ^10,11^. Paper is known to exhibit crack roughening^11^, a fact that can be checked by tearing a piece by hand and lead to self-affine roughness (see e.g. ref.^11–13^) and intermittent crack propagation^14–16^. Less known is that the energy dissipation is also detectable by Infra-Red (IR) imaging, as the material heats up under loading and in the presence of a defect the localized heating creates noticeable or easily measurable “hot spots”, with a clear temperature difference to the rest of the sample^11,17,18^. We combine the sample stress-strain curve with IR observations, and perform simultaneous Digital Image Correlation (DIC) analyses of the sample strain fields^19–21^ to look at the causality of the crack propagation or its relation to the maximum dissipation and to the peaks of the tensile stresses (as measured by the strain fields) during crack propagation and simultaneous roughening.

## Results

**Figure 1:**
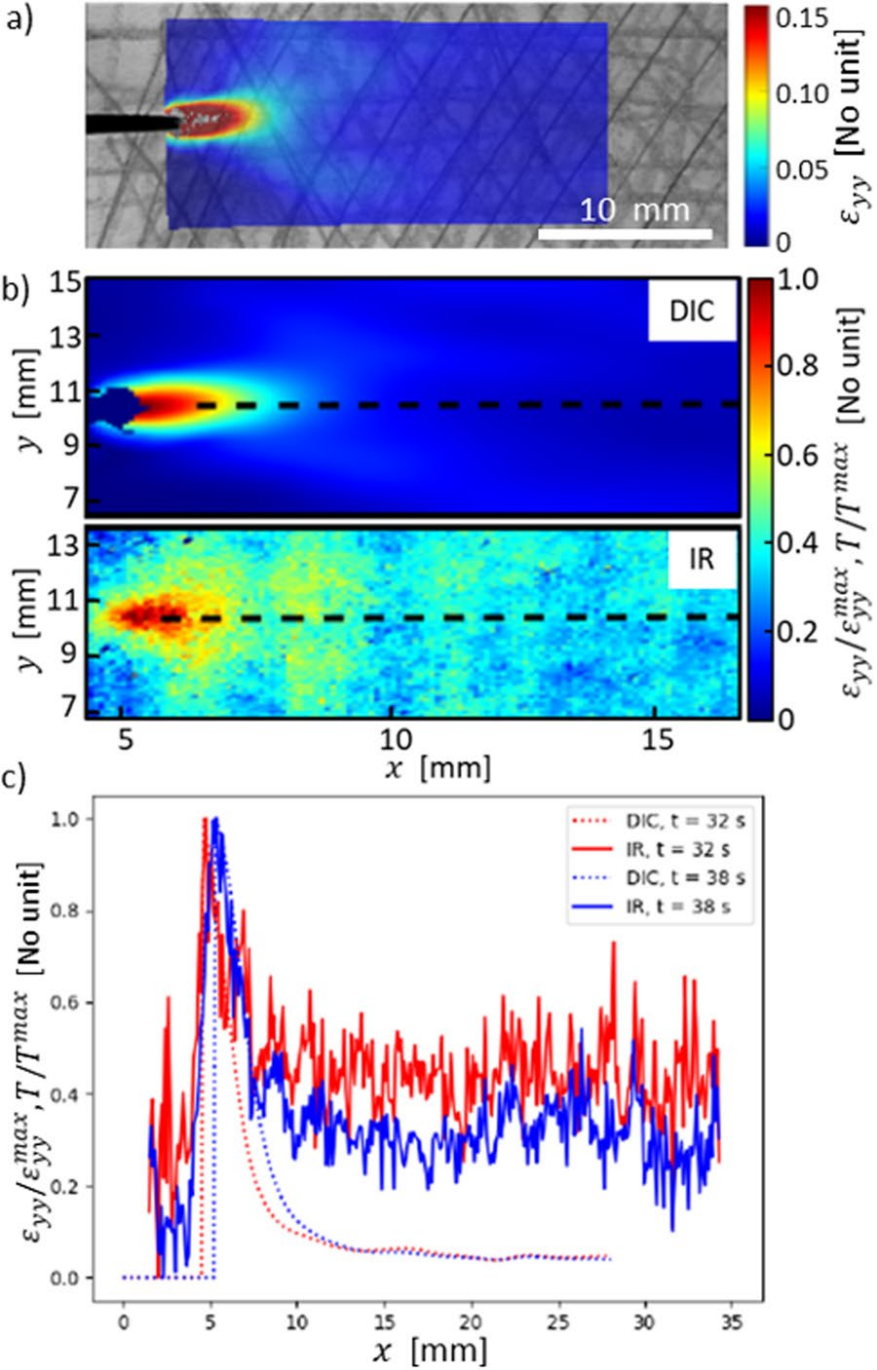
Snapshots at time *t* = 38 s show the spatial strain and temperature fields. (**a**) An example of an IR image, superimposed on the background. This temperature field shows the hot spot which has a temperature 0.4 °C higher than the background value of 22.0 °C. The coordinates are indicated, and one may see how the paper sample texture has been enriched for easier DIC computations. (**b**) DIC (*ε*_*yy*_) and IR fields at the same instance in time. (**c**) DIC and IR profiles parallel to the crack propagation direction at two instances of time.

In what follows, we discuss a typical experiment. The simultaneous measurement of the sample surface temperature fields and local displacements (see Methods section) allows to superimpose the measured fields on the top of each other and look for their simultaneous development with time in a tensile test. This is illustrated in Fig. 1, which shows the a) DIC and b) IR results and how they compare with each other. The temperature profile (Fig. 1c) of the sample shows an increase of the local temperature next to the crack tip at non-zero strain rates as expected. Local deformations are also at the largest in the proximity of the tip. Most importantly, the temperature rise shows some time into the test that localized “hot spots” are formed at some distance from the actual nominal crack tip, with a typical temperature increase of 0.4 °C compared to the (sample) background temperature of 22.0 °C. Similarly the maximum strain can rise upto 15%. Our main idea in what follows is to chart their dynamics in relation to the stress-strain curve and to the DIC-measured strain fields. Well beyond the neighborhood of the tip/FPZ the fields (strain *ε*, temperature change) decay to the background values.

**Figure 2:**
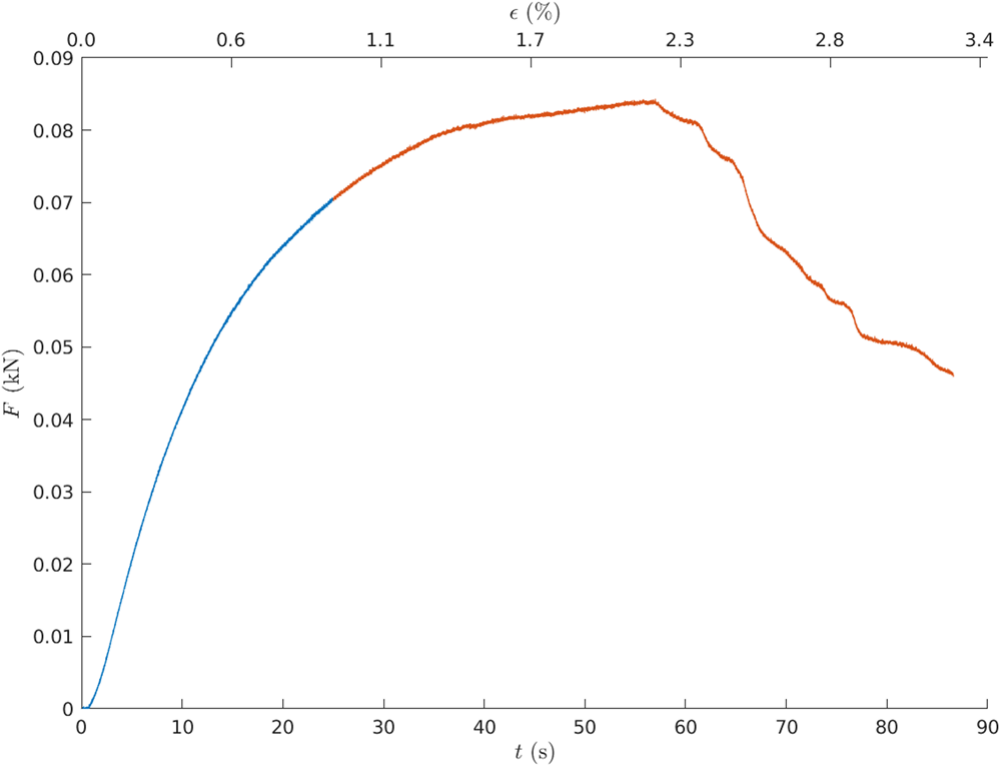
A typical stress-strain curve in a crack growth experiment obtained from the tensile testing machine. Here the constant displacement rate is *v* = 0.04 mm/s and *t* = 60 seconds corresponds to a total strain of *ε* = 2.3%. The blue color indicates a region where there is no crack growth observable by naked eye. The crack starts to advance in the red regime.

We use here and in what follows seconds instead of strain to underline the timescale of the hot spot development. The sample stress-strain curve in Fig. 2 shows the typical features of notched paper samples^11^. For small enough strains the behavior is roughly linearly elastic. The bending of the stress-strain curve around *t* = 20 s is a signature of the development of plastic deformation and irreversible strain. During this phase, the “hot spot” appears in the IR images and becomes a feature with details we discuss below. At around 60 seconds (corresponding to a strain of 2.3%) the peak stress *σ*_*c*_ is reached, and beyond that peak the hot spot is detectable until the final sample failure, which occurs at around 90 seconds. The last part of the stress-strain curve corresponds to stable crack propagation with the elastic compliance of the sample changing (decreasing) in a linear manner.

However, the crack already propagates slightly before this. Starting around *t* = 37 s there is a clear evidence that the crack has advanced. The effect of the crack propagation to the carried load of the sample is small compared to the propagation after *t* = 60 s.

**Figure 3:**
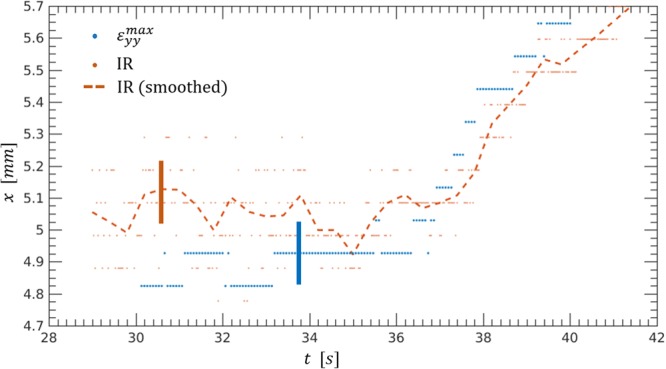
The x-coordinates of the hot spot, their running average and the position of the maximum strain show that the crack starts the stable propagation at *t* = 37 s. The thick vertical bars are the error bars corresponding to the discretization of the data which is the largest source of error.

The first main result of the IR to DIC comparison is illustrated in Fig. 3. The figure corresponds to the red part (*t* > 30 s) of the stress-strain curve of Fig. 2 right after the hot spot has first appeared. Three quantities or positions in the (*x*−) direction of the crack propagation are shown, the maximum tensile strain *ε*_*yy*_ from DIC, the instantaneous position of the hot spot, and a running average of its location. Up to about 37 seconds the spot stays slightly ahead of the maximum of the strain, and after that they start to move in unison (compare with Fig. 2). In general, they anticipate the actual crack tip and in particular the FPZ.

**Figure 4:**
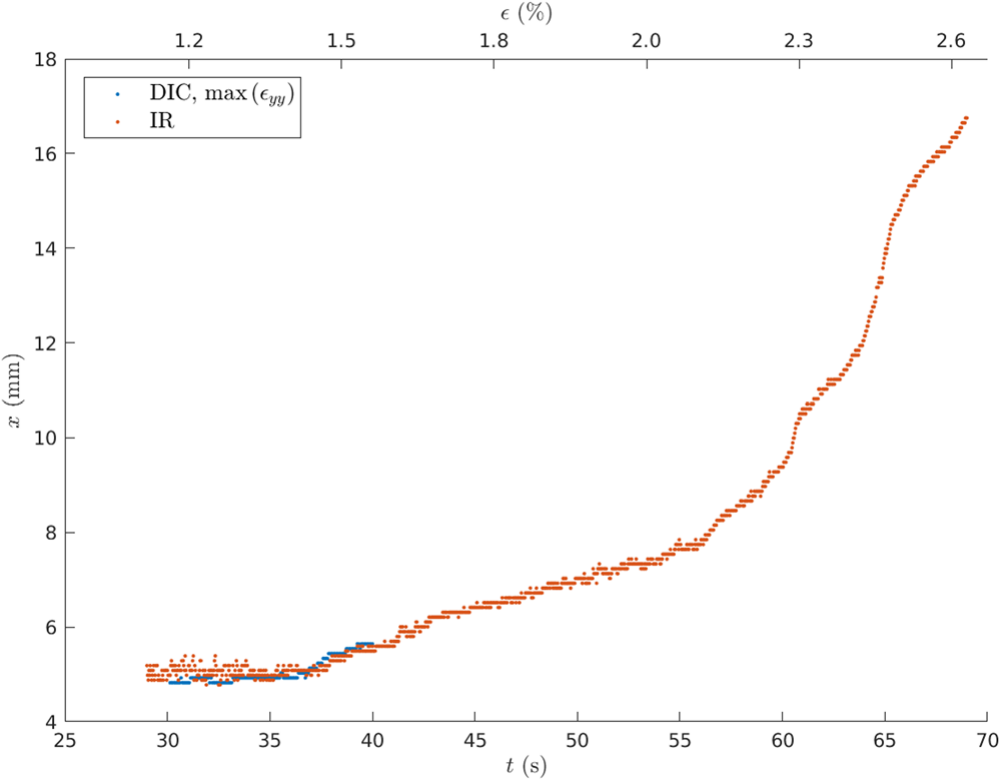
The full crack path (x vs. time) of the hot spot (red, IR) is available also beyond the time when DIC becomes unreliable (${\varepsilon }_{yy}^{max}$, blue). We notice again the acceleration in crack growth.

The later development during the test is shown in Fig. 4, up to close to 70 seconds. The figure follows the averaged hot spot and the strain maximum. Eventually the strain field, getting close to the peak of the stress strain curve, gets too diffuse for one to be able to define the maximum and its location. Up to this point, the hot spot location agrees with it within the accuracy of the measurements. The post-peak (60 seconds and beyond) behavior of the stress-strain curve (Fig. 2) is in agreement with the post-peak dynamics of the hot spot, which starts to move faster with time.

**Figure 5:**
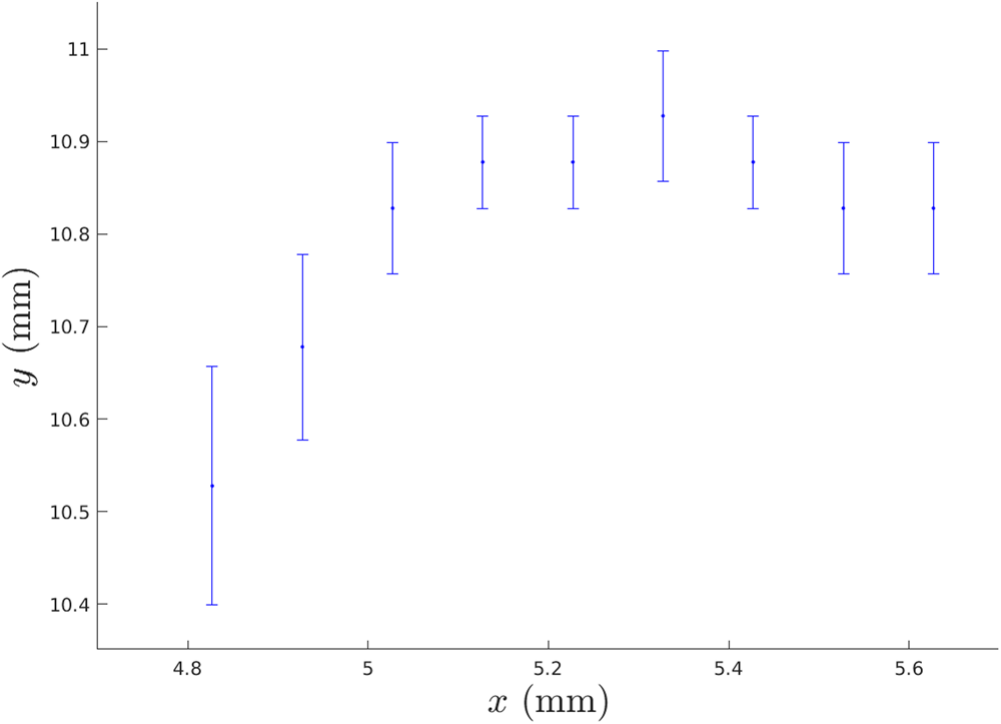
The crack location as the location of ${\varepsilon }_{yy}^{max}$ at various times shows some movement of the tip perpendicular to the direction of propagation. The location is the maximum of the vertical strain. The ~0.1 mm discretization in DIC is seen as steps in the figure. The y-coordinate is measured from the lower left corner of the DIC image area.

Figure 5 shows the transverse location of the crack tip determined from the DIC algorithm as maximum strain. During the initial 1 mm crack propagation in the x-direction, the transverse movement of the crack tip in the y-direction is 0.5 mm. In this small scale the transverse movement is significant corresponding to a 30 degree angle. Compared to sample size of 105 × 93 mm the transverse movement of 0.5 mm is insignificant and of the same order of magnitude as the hot spot size. Typical crack roughness in paper samples of this type is of the same order of magnitude^11^.

## Discussion

We have investigated by combined, simultaneous DIC and IR measurements the driving mechanisms in crack propagation, in the presence of a Fracture Process Zone in a typical paper fracture experiment. The maximum dissipation location can be measured by IR due to the local temperature rise and defined from the temperature fields. It in general agrees with the DIC indicated spot for maximum tensile stresses, i.e. the location of peak plastic deformation. These results are important, since they show that in the case of paper the intermittent dynamics of crack advancement are not independent, but they are instead intimately coupled to the dissipative processes well ahead (in space, in the direction of crack propagation) of the FPZ and the actual crack tip. This in turn is crucial in considering the roughening of the crack surfaces and the role of interface depinning in understanding crack dynamics. Our results show that there is a case in which cracks roughen, but via a different mechanism.

## Methods

**Figure 6:**
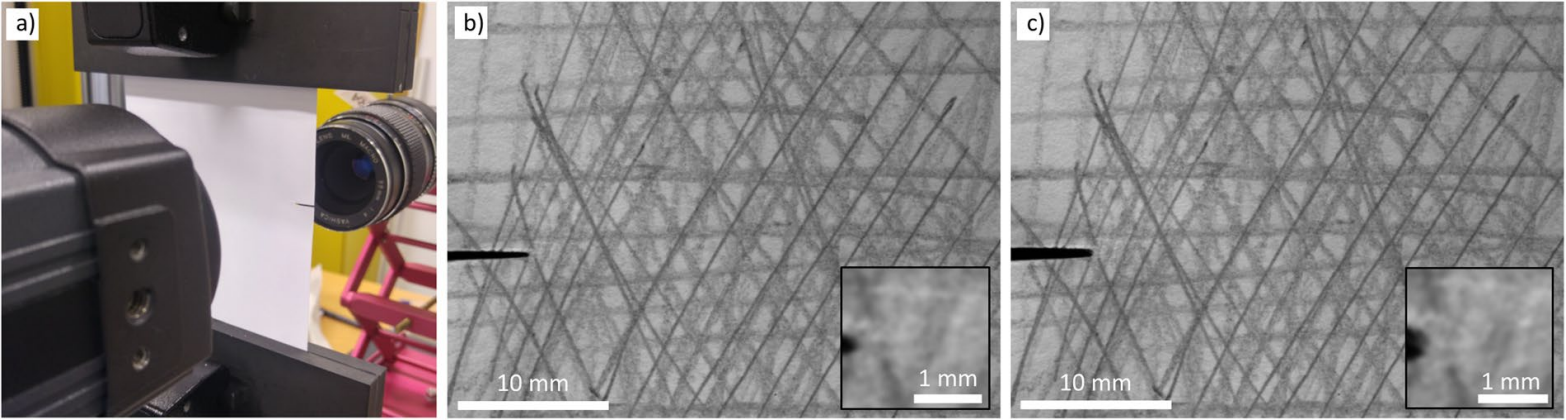
(**a**) The experimental setup showing the two simultaneous cameras on either side of the sample. (**b**) A raw image at *t* = 32 s. (**c**) A raw image at *t* = 38 s. The inset in panels (b) and (c) show magnification near the crack tip. It shows that the crack tip, as a whole, has not moved while there exists changes in texture due to strain.

The experimental setup consists of Instron E1000 tensile testing machine, Flir A320 thermal camera and Dalsa Genie HM1024 grayscale digital camera. The sample is standard 80 g/m^2^ copy paper, with pencil marks to facilitate the DIC computations (Fig. 6). The temperature is controlled with a standard laboratory facility air conditioning system with no humidity control. The sample width is 93 mm and it is clamped to the tensile testing machine such that the distance from the notch to the lower clamp is 50 mm and to the top clamp 55 mm, the total sample height being 105 mm between the clamps. As 10 mm is reserved for attaching in both ends the total sample length is 125 mm. The length of the initial notch is 10 mm. The origin of the coordinate system is set to the crack tip at *t* = 0 s by visual inspection. Images shown here are from later times where the crack tip has already propagated for a very small distance. Due to the diffuse nature of the FPZ in copy paper accurate crack tip determination is not feasible in particular at the early stages (here, 38 s) of an experiment.

The loading protocol is displacement controlled so that the velocity of the top clamp is *v* = 0.04 mm s^−1^ which corresponds to a strain rate of $\dot{\varepsilon }$ = 3.8 · 10^−4^ s^−1^. The tensile testing machine measures the force that is then converted to stress by dividing by the initial sample cross sectional area.

The thermal camera operates with a frequency of 20 Hz and an image area of 320 × 240 pixels and can detect temperature differences of 0.05 °C which corresponds to the noise level in the thermal data. The digital grayscale images are obtained with an image area of 1024 × 768 pixels and a frequency of 10 Hz. The grayscale images are processed with a Digital Image Correlation algorithm^22,23^ using a region of interest of 32 × 32 pixels.

The spatial accuracy is set in a way where a pixel in thermal imaging corresponds to 0.1 mm, which is eventually the same accuracy we obtain for strain fields. One region of interest in DIC is 32 × 32 pixels leading to 0.1 × 0.1 mm in spatial accuracy.

## Data Availability

The data used in this manuscript can be obtained from the authors with a reasonable request.

## References

[1] Alava MJ, Nukala PKKV, Zapperi S (2006). Statistical models of fracture. Adv. Phys..

[2] Måløy KJ, Santucci S, Schmittbuhl J, Toussaint R (2006). Local waiting time fluctuations along a randomly pinned crack front. Phys. Rev. Lett..

[3] Bares J, Barlet M, Rountree CL, Barbier L, Bonamy D (2014). Nominally brittle cracks in inhomogeneous solids: from microstructural disorder to continuum-level scale. Front. Phys..

[4] Bonamy D, Santucci S, Ponson L (2008). Crackling dynamics in material failure as the signature of a self-organized dynamic phase transition. Phys. Rev. Lett..

[5] Tanguy A, Gounelle M, Roux S (1998). From individual to collective pinning: Effect of long-range interactions. Phys. Rev. E.

[6] Koivisto J, Rosti J, Alava MJ (2007). Creep of a fracture line in paper peeling. Phys. Rev. Lett..

[7] Ponson L (2009). Depinning transition in the failure of inhomogeneous brittle materials. Phys. Rev. Lett..

[8] Bazant ZP (2004). Scaling theory for quasibrittle structural failure. Proc. Natl. Acad. Sci..

[9] Alava MJ, Nukala PKVV, Zapperi S (2008). Role of disorder in the size scaling of material strength. Phys. Rev. Lett..

[10] Kettunen H, Niskanen K (2006). Microscopic damage in paper. Part I: Method of analysis. J. Pulp Pap. Sci..

[11] Alava MJ, Niskanen KJ (2006). Physics of paper. Reports on Prog. Phys..

[12] Salminen LI, ALava MJ, Niskanen K (2003). Analysis of long crack lines in paper webs. Eur. Phys. J. B.

[13] Alava, M. J., Nukala, P. K. V. V. & Zapperi, S. Morphology of two dimensional fracture surfaces. *J. Stat. Mech*. L10002 (2006).

[14] Santucci S, Vanel L, Ciliberto S (2004). Subcritical statistics in rupture of fibrous materials: Experiments and model. Phys. Rev. Lett..

[15] Santucci S, Cortet P-P, Deschanel S, Vanel L, Ciliberto S (2006). Subcritical crack growth in fibrous materials. Europhys. Lett..

[16] Stojanova M, Santucci S, Vanel L, Ramos O (2014). High frequency monitoring reveals aftershocks in subcritical crack growth. Phys. Rev. Lett..

[17] Toussaint R (2016). How cracks are hot and cool: a burning issue for paper. Soft Matter.

[18] Koivisto J, Ovaska M, Miksic A, Laurson L, Alava MJ (2016). Predicting sample lifetimes in creep fracture of heterogeneous materials. Phys. Rev. E.

[19] Hild F, Roux S (2006). Digital image correlation: from displacement measurement to identification of elastic properties – a review. Strain.

[20] Miksic, A., Koivisto, J. & Alava, M. J. Statistical properties of low cycle fatigue in paper. *J. Stat. Mech*. P05002 (2011).

[21] Koivisto J, Dalbe M-J, Alava MJ, Santucci S (2016). Path (un)predictability of two interacting cracks in polycarbonate sheets using digital image correlation. Sci. Reports.

[22] Kybic J, Thévenaz P, Nirkko A, Unser M (2000). Unwarping of unidirectionally distorted EPI images. IEEE Transactions on Med. Imaging.

[23] Rosti J, Koivisto J, Laurson L, Alava MJ (2010). Fluctuations and scaling in creep deformation. Phys. Rev. Lett..

